# Enhancing Exchange-Traded Fund Price Predictions: Insights from Information-Theoretic Networks and Node Embeddings

**DOI:** 10.3390/e26010070

**Published:** 2024-01-12

**Authors:** Insu Choi, Woo Chang Kim

**Affiliations:** Department of Industrial and Systems Engineering, Korea Advanced Institute of Science and Technology, Daejeon 34141, Republic of Korea; jl.cheivly@kaist.ac.kr

**Keywords:** mutual information, transfer entropy, machine learning, node embedding, centrality measure, explainable artificial intelligence (xAI)

## Abstract

This study presents a novel approach to predicting price fluctuations for U.S. sector index ETFs. By leveraging information-theoretic measures like mutual information and transfer entropy, we constructed threshold networks highlighting nonlinear dependencies between log returns and trading volume rate changes. We derived centrality measures and node embeddings from these networks, offering unique insights into the ETFs’ dynamics. By integrating these features into gradient-boosting algorithm-based models, we significantly enhanced the predictive accuracy. Our approach offers improved forecast performance for U.S. sector index futures and adds a layer of explainability to the existing literature.

## 1. Introduction

Classifying listed companies into sectors offers a comprehensive perspective that is pivotal for constructing financial portfolios. The significance of the sector index as a primary boundary in classification systems, encompassing industry groups and sub-industries, is well established in the literature. This sectorial approach grants investors unique advantages, allowing them to harness specific sector opportunities, balance underrepresented sectors, and adopt active investment strategies while considering macroeconomic shifts, momentum, and other crucial factors [1]. 

However, while many studies have delved into sector indices and ETFs, Leung and Zhao (2021) rightly observed a research gap: there is a limited exploration of the price dynamics comparison between sector indices and ETFs. Research examining the nonlinear dependencies and causalities within sector indices, especially from an information-theoretic viewpoint, remains sparse [2].

Our contribution thus stands at the intersection of these identified gaps. The novelty in our approach lies in two key aspects:

We provide a fresh perspective by delving into the nonlinear network topologies of sector indices in the U.S. market, a dominant global player. This exploration unravels intricate nonlinear information exchanges and flows, showcasing a departure from traditional linearity-based studies.

Also, our study harnesses the potential of these insights to craft predictive models. We tap into their unique predictive power by utilizing a range from short-term (20-day window) to long-term (240-day window) nonlinear dependency and causal networks, incorporating centrality-based measures and novel node embedding techniques.

The manuscript is methodically structured, offering readers a clear progression through our research. Section 2 begins by setting our study within the context of the existing literature and also presenting our dataset. Section 3 offers a thorough explanation of our research methodology, which is intricately linked to the statistical properties of our data. In Section 4, we embark on the construction and analysis of networks, focusing on U.S. sector indices’ returns across varied time frames, while diving deeply into the inherent nonlinear dependencies and causalities. Section 5 serves as a reflective space, discussing the real-world implications of our findings, especially in terms of node-level network measures, the innovative use of node embeddings, and the demonstrable success of our predictive models. Finally, Section 6 wraps up our discourse, providing concluding thoughts on our contributions and hinting at promising avenues for future research.

## 2. Data and Methodology

### 2.1. Prior Research

In finance, sector and industry research incorporates varied macroeconomic views. Krause and Tse (2013) identified a consistent pattern of price discovery moving from the U.S. to Canada in securities, with bidirectional volatility spillovers [3]. Hernandez et al. (2015) pointed out a one-way time lag relationship between the IT-computer and medicine-biotechnology sectors, with the former influencing the latter’s price and return values [4]. Dutta (2018) revealed a persistent correlation between oil and stock market volatility indices [5]. Shahzad et al. (2018) used the Q-Q method to demonstrate an asymmetric negative relationship between credit and markets across industries, dependent on the nature and extent of stock market shocks [6]. Khan et al. (2020) confirmed short- and medium-term causality between the Financial and Economic Attitudes Revealed by Search (FEARS) and stock returns, with a stronger correlation in specific sectors [7]. Matos et al. (2021) discovered that early pandemic death cycles in Italy, followed by similar patterns globally, were indicators of disruptions in the U.S. stock market, with the energy sector reacting first to the pandemic [8]. Wan et al. (2021) suggested that solid media sentiment towards a particular company in an industry could lead to a significant shift in media sentiment towards related companies [9]. Shahzad et al. (2021) found that network structures and spillovers greatly vary with market conditions [10]. Choi and Kim conducted an empirical analysis on politically-themed stocks in South Korea, creating networks based on these stocks influenced by political figures [11]. Jin and Guo (2021) showed that since 2013, specific sector indices like consumption, industry, and real estate have been leading corresponding macroeconomic variables in the U.S. stock market [12]. Finally, Mensi (2022) found that sectors like oil, gold, financials, utilities, communication services, consumer staples, and healthcare are net recipients of spillovers, while other sectors are net contributors, determined through methods like the time-frequency spillover method, wavelet method, and the DCC-GARCH model [13].

In financial product price and return prediction, Beer et al. (2020) delved into the innovative territory of deep quantum neural networks for financial forecasting [14]. Chen, Zhang, and Lou (2020) developed a sophisticated model for stock price prediction using a hybrid deep learning approach, combining an attention mechanism with a multilayer perceptron and a bidirectional long short-term memory neural network [15]. Chen and Zhou (2020) introduced a stock prediction model that synergizes genetic algorithm feature selection with a long short-term memory neural network [16]. Althelaya, Mohammed, and El-Alfy (2021) enhanced stock market forecasting by integrating deep learning with a multiresolution analysis, demonstrating the efficacy of combining varied analytical methods [17]. Aldhyani and Alzahrani (2022) crafted a novel framework using deep learning techniques, marking a significant stride in computational finance [18]. 

Building upon the foundation established by the research, our study carves out a distinctive niche in sector- and industry-based research. While previous studies have predominantly operated within a linear paradigm, our work diverges by incorporating a nonlinear perspective. We not only challenge the prevalent focus on linear correlations or causalities between sectors and industries, but also enrich the discourse by introducing and utilizing the network theory. By adopting this approach, we delve deeper into the intricate nonlinear connections and dependencies within and between sectors. Furthermore, our integration of the network theory offers a fresh lens through which we can decode the complex interplay and dynamics of these sectors. Thus, our research expands the analytical boundaries of sector-based financial research and provides a novel toolkit to better understand and predict sector behaviors and interactions in today’s intricate financial landscape.

### 2.2. Data

We used Standard and Poors’ U.S. sector index ETFs’ price and trading volume data as the experimental data. There are eleven sectors in the sector Standard and Poor’s Depository Receipt (SPDR) ETFs: Materials (XLB), Communications Services (XLC), Energy (XLE), Financials (XLF), Industrials (XLI), Technology (XLK), Consumer Staples (XLP), Real Estate (XLRE), Utilities (XLU), Health Care (XLV), and Consumer Discretionary (XLY). In this paper, we only used the data from nine sector indices (XLB, XLE, XLF, XLI, XLK, XLP, XLU, XLV, and XLY), because those nine sector indices, from the beginning, were listed on the New York Stock Exchange (NYSE) Arca Exchange on 16 December 1998, but the other two were not. XLRE was listed on 8 October 2015 at NYSE Arca Exchange, and XLC was listed on 19 June 2018 at NYSE Arca Exchange. Those two indices have relatively fewer data; thus, we cannot conduct a network analysis and predict their fluctuation at the same condition. The experimental period is from January 2010 to September 2022. This experimental period is twelve years and nine months long, including a total of 51 quarters and 153 months. In the case of the predictive experiment, we designated the period from January 2010 to December 2018 (about 70% of the whole dataset) as the training and validation set and designated the period from January 2019 to September 2022 (about 30% of the whole dataset) as the testing set. The target data are only the return data of the nine U.S. sector index ETFs. Table 1 provides an overview of the datasets employed throughout our research.

## 3. Methodology

Based on Shannon’s entropy [19] concept, mutual information and transfer entropy serves as a nonparametric methodology to verify information exchange between pairs of variables.

Data assumptions such as normality, stationarity, and linearity should be preceded by general dependencies and causal relationships represented like Granger causality [20,21]. However, it is known that the nature of stock return-based data usually only satisfies some of these properties. Therefore, we used theories of econopyhsics and information theories that can be used without the above assumptions. To use these theories, we can consider nonlinear relationships between objectives to measure dependencies and causal relationships. Accordingly, we used the concept of mutual information (MI), first proposed by Shannon, and transfer entropy (TE), proposed by Schreiber (2000) [22]. These are entropy-based measures. Specifically, in this study, we used normalized mutual information (NMI) and transfer entropy (TE) based on Shannon entropy with a permutation test for threshold network construction (Boba et al., 2015) [23].

### 3.1. Mutual Information

#### 3.1.1. Mutual Information

Mutual Information (MI) is a measure that captures the shared information between two variables, indicating their statistical interdependence. In the field of information theory, the behavior of a system, say System X, is understood through its probability distribution p(x) and logarithm value of p(x). Based on this idea, the Shannon entropy is as follows:

(1) $$H(X)=-\sum_{x\in X}p(x){log}_{2}p(x).$$

Shannon entropy quantifies the information required to identify random values from a discrete distribution. When two subsystems, X and Y, are present in a state of the system, their combined probability distribution is represented by a joint probability.

(2) $$H\left(X,Y\right)=-\sum_{x\in X,y\in Y}p\left(x,y\right){log}_{2}p\left(x,y\right).$$

Finally, we can define MI as the quantity of identifying the interaction between subsystems.

(3) I(X,Y)=H(X)+H(Y)−H(X,Y)

Mutual Information (MI) has been widely utilized in finance for network analysis across various stock exchanges. It’s been instrumental in developing networks and selecting portfolios, mainly using short-term data in different markets, and examining market behaviors during significant changes or events. This approach provides insights into market dynamics and investor sentiments in diverse economic contexts [24,25,26,27,28,29,30].

#### 3.1.2. Normalized Mutual Information (NMI)

One of MI’s disadvantages is that it is hard to compare results from the MI derived from different data. Because the domain of MI is always finite for the discrete random variables, the maximum value of the MI is not constant. In other words, this means that it is hard to compare the statistical dependence derived from different datasets. Therefore, we used NMI to compare politically themed stock networks within the same range [0,1]. Since there are several normalized variants of NMI, their properties are slightly different. In this study, we used NMI with a minimum of two entropies, as shown in (4), because the normalization version of mutual information measures should be based on the least upper bound, min(H(X),H(Y)). Using NMI with a minimum of two entropies ensures that the maximum attainable value of NMI is one. This version of NMI is irrespective of the dimensions of two discrete variables and the marginal probabilities [31,32,33,34,35] (Kvålseth, 1987; Banerjee et al., 2005; Kraskov et al., 2005; Vinh et al., 2010; Sarhrouni et al., 2012; Kvålseth, 2017).

(4) $$NMI(X,Y)=\frac{I(X,Y)}{min(H(X),H(Y))}$$

### 3.2. Transfer Entropy (TE)

Transfer Entropy (TE), based on Shannon entropy and mutual information concepts, is a non-parametric metric for quantifying information transfer between two variables. Unlike Granger causality, which is prediction-oriented, TE focuses on reducing uncertainty, measuring how one variable clarifies the future of another beyond its own past contributions. TE stands out as a model-free approach for identifying causal links in dynamic systems, particularly useful in finance for analyzing connections between various financial entities and market dynamics. This method is renowned for its ability to efficiently pinpoint sources and targets in causal relationships [36].

Transfer Entropy has been a key tool in financial research to explore causal relationships. Studies have examined the connections between credit default swap and bond markets, the causal links among international financial firms, the interplay between exchange rates and stock prices in emerging economies, and the information flow in U.S. equity and commodity markets. This method has proven effective in understanding both internal and cross-market dynamics, demonstrating its versatility in different financial contexts [37,38,39,40].

Based on the concepts mentioned earlier related to entropy, conditional entropy quantifies the amount of information needed to describe the outcome of a random variable, X, given that the value of another random variable, Y, is known. Here, the conditional entropy of X given Y can be expressed as

(5) $$H\left(X|Y\right)=-\sum_{x\in X,y\in Y}p\left(x,y\right){log}_{2}\frac{p(x,y)}{p(y)}.$$

**Figure 1:**
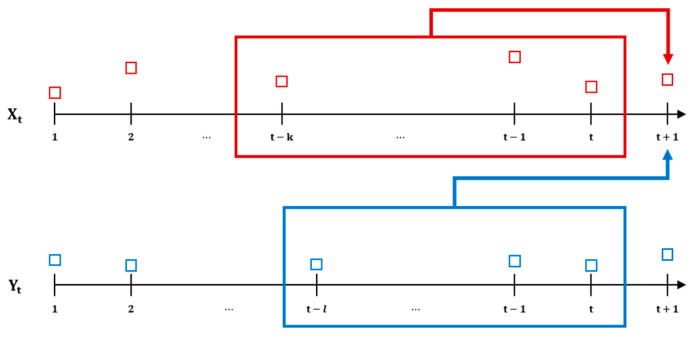
Schematic representation of transfer entropy (I. Choi. 2021).

It can be interpreted as the uncertainty about Y when X is known, or as the expected number of bits needed to describe X when Y is known to both the encoder and the decoder. Based on the above definition, we can define the general form of $\left(k,l\right)$-history TE between two time series, $X_{t}$ and $Y_{t}$, for $x_{t}^{\left(k\right)}=(x_{t},\ldots ,x_{t-k+1})$ and $y_{t}^{\left(l\right)}=(y_{t},\ldots ,y_{t-l+1})$. The general $\left(k,l\right)$-history transfer entropy can be expressed as follows (Bossomaier et al., 2016):

(6) $$\begin{array}{l} {TE}_{Y\to X}^{\left(k,l\right)}\left(t\right)=H\left(X_{t+1}|X_{t},\ldots ,X_{t-k+1}\right)-H\left({X_{t+1}|X}_{t},\ldots ,X_{t-k+1},Y_{t},\ldots ,Y_{t-l+1}\right)\\ =\sum_{i}p\left(x_{t+1},x_{t}^{\left(k\right)},y_{t}^{\left(l\right)}\right){log}_{2}p\left(x_{t+1}\right|x_{t}^{\left(k\right)},y_{t}^{\left(l\right)})-\sum_{i}p\left(x_{t+1},x_{t}^{\left(k\right)},y_{t}^{\left(l\right)}\right){log}_{2}p\left(x_{t+1}\right|x_{t}^{\left(k\right)})\\ \;\;\;\;\;\;\;\;\;\;\;\;\;\;\;\;=\sum_{i}p(x_{t+1},x_{t}^{\left(k\right)},y_{t}^{\left(l\right)}){log}_{2}\frac{p(x_{t+1}|x_{t}^{\left(k\right)},y_{t}^{\left(l\right)})}{p(x_{t+1}|x_{t}^{\left(k\right)})}, \end{array}$$

where $i=\left\{x_{t+1},x_{t}^{\left(k\right)},y_{t}^{\left(l\right)}\right\}$. ${TE}_{Y\to X}^{\left(k,l\right)}\left(t\right)$ is non-negative, and we can drop the time dependency argument, t, for stationary processes. ${TE}_{Y\to X}^{\left(k,l\right)}\left(t\right)$ represents the information about the future state of $X_{I}$, which can be obtained by subtracting information retrieved from only $X_{t}^{\left(k\right)}$ from the information gathered from both $X_{t}^{\left(k\right)}$ and $Y_{t}^{\left(l\right)}$. The schematic representation of transfer entropy is shown in Figure 1.

In this study, we focused on the TE under the following conditions of two lags, k=l=1. These settings for lags are typically chosen as they align with the principles of the weak form of the Efficient Market Hypothesis (EMH) and the notion that stock prices follow a random walk pattern. [39,40]. Then, we can express the equation of (1,1)-history TE as follows:

(7) $${TE}_{Y\to X}^{\left(1,1\right)}\left(t\right)=\sum_{i}p\left(x_{t+1},x_{t},y_{t}\right){log}_{2}\frac{p\left(x_{t+1}|x_{t},y_{t}\right)}{p\left(x_{t+1}|x_{t}\right)}=\sum_{i}p(x_{t+1},x_{t},y_{t}){log}_{2}\frac{p(x_{t+1},x_{t},y_{t})p(x_{t})}{p(x_{t+1},x_{t})p(x_{t},y_{t})},$$

where $i=\left\{x_{t+1},x_{t},y_{t}\right\}$.

### 3.3. Test for Obtaining p-Values of MI and TE

In recent research, transfer entropy has often been explored without considering the finite-size effects arising from sample variations. In this study, we adopt a nuanced approach by integrating the permutation test alongside transfer entropy to effectively mitigate these finite-size effects [23,36]. The strength of permutation tests lies in their non-parametric nature, necessitating minimal assumptions and making them particularly adept at discerning statistical significance, especially when the data-generating mechanism remains elusive. To ensure robustness, we introduced a shuffling mechanism for each element in the time series, deriving mutual information and transfer entropy complemented by *p*-values. Our methodological choice of conducting 1000 shuffles (M = 1000) to compute ETE values underscores our commitment to precision. This novel integration, encapsulating both permutation tests and transfer entropy, is our distinct contribution to the literature, offering insights with heightened accuracy.

### 3.4. Network Analysis

Network analysis offers a unique lens through which we can gain insights into complex social phenomena by representing them as interconnected systems. This approach not only simplifies the depiction of interactions but also provides a structured framework for understanding the intricate dynamics between different entities. In the context of our research, we view the entropy-based causal relationships among exchange rates as a web of interactions between sector ETFs. By doing so, we unlock the potential to visualize and delve deeper into the intricate ties binding these sector ETFs.

For our study, we leveraged various network topology metrics, transforming them into features for our predictive models. These metrics serve as crucial indicators, helping us navigate the vast network landscape and understand our dataset’s underlying patterns.

#### 3.4.1. Network Theory

Nodes and links are the fundamental components of network theory. As a subject component of a network, a node functions as an interactive agent. A link or connection between two subjects is also an interaction between them. The network type can be classified according to the connection’s characteristics. Networks can be classified as directional or non-directional depending on whether they have a direction. Moreover, if the network is weighted, it is known as a weighted network; otherwise, it is known as a binary network.

Graphs and matrices are used to represent networks. Both systems have advantages in terms of mathematical processing and visual explanation. Using a graph format is a means to depict a network and intuitively show its shape by giving nodes and links shapes, colors, sizes, labels, and arrows. A matrix format describes network attributes as a matrix, often called adjacency matrix.

We used the weighted-undirected graph for MI and the directed-weighted graph for TE. Then, we used the *p*-value from the permutation tests as a threshold for deciding connections between two U.S. sector index ETFs’ log-returns and trading volumes’ rates of changes. In other words, the *p*-values of MI and TE determine the linkages in the created directed weighted network. Our *p*-value-based threshold value was 0.1, one of the conventional statistics values.

In detail, each edge $\left(u,v\right)\in E$ was attributed a $w\left(u,v\right)$ calculated from one of our statistical dependency measures and an associated *p*-value, $p\left(u,v\right)$.

To construct a *p*-value Threshold Tree, we define a *p*-value criterion under which an edge is considered statistically significant. For a commonly used significance level of α=0.05, our construction rule might be articulated as

(8) $$E_{\alpha }=\{\left(u,v\right)\in E|p\left(u,v\right)<\alpha \}$$

#### 3.4.2. Centrality Measures

Our data were collected from observations within the same system over time. We analyzed topological measures of an evolving network at regular time intervals, yielding time-ordered sequences of topological observations. Our focus was primarily on centrality measures, which are quantified by applying a real-valued function to the vertices of a graph, aiming to rank nodes based on their significance.

The concept of “importance” in a network can be interpreted in various ways, leading to different centrality definitions. One approach conceptualizes importance in terms of network flow or transfer, categorizing centralities based on the type of flow they emphasize. Alternatively, importance can be seen as a node’s contribution to network cohesion, leading to centralities that assess cohesiveness.

Different centrality measures consider the number of paths passing through a node, varying in their definition and counting of relevant paths. This approach allows for a classification spectrum ranging from centralities concerned with short paths (like degree centrality) to those involving longer or infinite paths (such as eigenvector centrality). Other measures, like betweenness centrality, focus on a node’s role in overall network connectivity. The centrality measures used in our study are detailed in Table 2, with their respective citations ranging from [41,42,43,44,45,46,47,48,49,50,51,52,53,54,55,56,57,58,59,60,61,62,63,64,65,66,67,68].

#### 3.4.3. Node Embedding Algorithm and Dimensionality Reduction

Understanding the intricate relationships between financial assets is of paramount importance. Traditional approaches, however, often miss the nuanced, nonlinear connections inherent within networks of assets, such as U.S. sector index ETFs. This is where node embeddings come to the fore, offering a fresh perspective that traditional methods may overlook.

Node embeddings convert the intricate attributes and relationships of nodes within a network into representative vectors. These vectors encapsulate essential structural details and node-specific features, furnishing us with enhanced capabilities for analysis and prediction. In our research, we leaned into these advantages, focusing specifically on understanding how features derived from node structures within our constructed network could aid in predicting the movement of nine U.S. sector index ETFs.

We harnessed the power of two notable node embedding techniques: Role2vec [69] and FEATHER [70]. While both techniques excel at capturing essential node information, they differ subtly in their focuses. Role2vec zeroes in on the structural characteristics of nodes, and FEATHER brings to light the attributes specific to each node. By employing both, we ensured a comprehensive grasp of the diverse facets of the network, from its broader architecture to the individual attributes of its constituents.

To refine our approach, we adopted 1024-dimensional embeddings and distilled them into more manageable 32-dimensional vectors. This refinement was achieved using UMAP [61], a technique renowned for preserving global structural information while having its roots firmly planted in Riemannian manifold and topological data analysis.

After deriving the node embeddings in our study, we further integrated them into our prediction framework. Specifically, we based our predictions on networks influenced by both mutual information and transfer entropy. This deliberate integration into the prediction problem enabled us to tap into the information-theoretic network structures.

##### Role2vec [69]

Ahmed et al. (2018) presented the Role2Vec framework, which employs the flexible concept of attributed random walks and serves as the foundation for leveraging random walks. Their proposed framework expands the applicability of these methods to transductive and inductive learning, as well as graphs with attributes (if available). This is accomplished by acquiring functions that are applicable to new nodes and graphs [69].

Role2vec focuses on learning role-based graph embeddings. The core idea is to learn a mapping of nodes to roles in the graph, and then learn embeddings for these roles. 

##### FEATHER [70] 

FEATHER, introduced by Rozemberczki and Sarkar (2020), is a flexible concept of characteristic functions defined on graph vertices to characterize the distribution of vertex features at multiple scales. FEATHER is a computationally efficient algorithm for calculating a particular variant of these characteristic functions in which the probability weights are defined as the transition probabilities of random walks [70].

Rozemberczki and Sarkar (2020) argued that the extracted features are useful for machine learning tasks at the node level. The pooling of these node representations yields compact graph descriptors that can serve as features for graph classification algorithms. They also demonstrated that FEATHER describes isomorphic graphs using the same representation and is resistant to data corruption analytically [70].

##### UMAP [71] 

UMAP is a dimensionality reduction technique that is well suited for visualizing high-dimensional datasets. Developed by McInnes, Healy, and Melville in 2018, UMAP operates based on Riemannian geometry and algebraic topology principles [71].

At its core, UMAP constructs a high-dimensional graph representation of the data and subsequently optimizes a low-dimensional version of this graph to produce a dimension-reduced representation. The method starts by approximating the data’s manifold by using a fuzzy simplicial set. The next step involves finding a low-dimensional representation of this set.

UMAP’s foundational mathematics relies on three main aspects:

1. **Topological Data Analysis:** Used to understand the high-dimensional structure of the data.
2. **Fuzzy Simplicial Sets:** Used to approximate the manifold the data resides on, providing both local and global preservation.
3. **Riemannian Geometry:** Used to accurately measure distances and maintain data relationships.

Our research employed UMAP to condense the 1024-dimensional vectors obtained from our node embeddings down to a more manageable 32-dimensional space. This reduction was imperative not only for visualization but also for enhancing computational efficiency without significantly compromising the structural integrity of our dataset. The robust foundation of UMAP in topological data analysis ensured that the global structure of our data was retained, making the resulting low-dimensional embeddings particularly insightful for subsequent analyses.

Integrating node embeddings is not just a technical addition, but a revolutionary step in bridging the identified research gaps. As our abstract suggests, we aim to bring a layer of explainability that is absent in the existing literature. Node embeddings, especially using Role2vec and FEATHER, allow us to achieve this. We transform abstract financial relationships into tangible, quantifiable data points by converting nodes and their intricate relationships into vectors. This paves the way for integrating these insights into predictive models that forecast with higher accuracy and provide deeper insights into the underlying dynamics.

While many studies have delved into sector indices and ETFs, our adoption of node embeddings elevates our research by emphasizing prediction and understanding. The resulting models are not black boxes, but interpretable tools that shed light on the intricate web of relationships within the U.S. sector index ETFs, marking a significant advancement in financial network analysis.

### 3.5. Machine Learning Algorithms and xAI (eXplainable Artificial Intelligence) Techniques

We mainly used the most frequently used basic machine learning models to predict index sector ETFs’ returns despite there being many state-of-the-art models with good performance. For example, although many studies have shown that recurrent neural networks and gradient-boosting algorithms based on them perform very well in various areas, this study aims to extract features that can be used in all machine learning models through a nonlinear measure-based network analysis to see their effects. Accordingly, we tried to confirm the performance of well-known machine learning techniques. We used three tree-based machine learning algorithms: XGBoost, LightGBM, and CatBoost [72,73,74]. We used those three prominent models for the following reasons:

1. **Interpretability**: Tree-based models, at their core, make decisions based on certain conditions, making them more interpretable than many deep learning models. This interpretability is vital in financial sectors, where understanding the reasons behind predictions can be as critical as the predictions themselves.
2. **Handling of Mixed Data Types**: Financial datasets often consist of numerical and categorical data. Tree-based models like CatBoost are particularly effective at handling categorical variables without extensive preprocessing.
3. **Automatic Feature Selection**: These models inherently perform feature selection. As a result, they can identify and prioritize the most essential features, which is particularly useful in financial datasets with potentially redundant or less impactful variables.
4. **Resistance to Overfitting**: With techniques such as gradient boosting and regularization in models like XGBoost and LightGBM, tree models exhibit resistance to overfitting, especially when appropriately tuned.
5. **Flexibility**: These models can easily capture nonlinear relationships in the data, which is common in financial time series data. Traditional linear models might not capture this nonlinearity as quickly.
6. **Efficiency and Scalability**: Models like LightGBM and CatBoost have been designed with efficiency in mind. They can handle large datasets, making them suitable for comprehensive financial data.
7. **Consistency in Results**: While deep learning models like RNNs can be potent, they require more meticulous fine-tuning and can sometimes produce inconsistent results due to their complex architectures. In contrast, tree models, once well-tuned, can provide more consistent predictions.
8. **End-to-End Modeling**: These models do not necessarily require extensive data preprocessing or normalization, making the modeling process more straightforward and sometimes more accurate since no information is lost in preprocessing.

#### 3.5.1. XGBoost

XGBoost (Chen and Guestrin, 2016) [72] is an algorithm that uses the boosting gradient technique proposed by Friedman (2001) [75]. XGBoost is an ensemble algorithm utilizing gradient tree boosting to enhance classifiers in a sequential manner. Its primary benefit lies in scalability across various situations, making it a highly popular choice for regression tasks.

(9) $${\hat{y}}_{i}=\sum_{k=1}^{K}f_{k}(x_{i}),f_{k}\in F$$

(10) $$Z=\sum_{i}l({\hat{y}}_{i},y_{i})+\sum_{k}\Omega (f_{k})=\sum_{i}l({\hat{y}}_{i},y_{i})+\sum_{k}(\gamma T+\frac{1}{2}\lambda ∥w{∥}^{2})$$

Equation (9) is an expression representing the ensemble model of the tree, F is the collective space of all possible classification and regression trees (CART). At this time, the final prediction is made by summing and comparing the scores of each leaf. Equation (10) is a normalized objective function of the XGBoost model. $l({\hat{y}}_{i},y_{i})$ is a differentiable convex loss function that measures the difference between the predicted and target values, and it is also a normalization term, and $\Omega (f_{k})$ is a CART function that prevents overfitting problems by smoothing the final learned weights by adjusting the complexity of the model. γT represents the number of leaves in CART, and $\frac{1}{2}\lambda ∥w{∥}^{2}$ represents the score assigned to the leaves in CART.

#### 3.5.2. Light Gradient Boosting Machine (LightGBM)

LightGBM, developed by Ke et al. in 2017 [73], is a gradient-boosting machine learning model that incorporates gradient-based one-side sampling (GOSS) and exclusive feature bundling (EFB) to handle variables efficiently. Its unique vertical growth structure makes it more accurate and efficient than other machine learning approaches.

(11) $$Y_{t}=\sum_{t=1}^{T}f_{t}(x)$$

In Equation (11), $f_{t}(x)$ is a tree, and its objective function is estimated using Newton’s method.

#### 3.5.3. CatBoost

CatBoost, introduced by Prokhorenkova et al. in 2018, is a gradient boosting algorithm focused on categorizing data. It stands out in handling categorical features through sequential boosting and decision tree-based techniques. The trees in CatBoost are created by grouping similar instances within the learning dataset, contributing to its superior performance compared to other gradient boosting methods.

#### 3.5.4. SHAP (Shapley Additive Explanation)

Machine learning shows promise in time series prediction but often lacks explanatory power. Addressing this, Lundberg and Lee (2017) introduced the SHAP method, enhancing interpretability across various machine learning models [76]. SHAP, based on the Shapley value from game theory [77], is a key approach in explainable AI (xAI), elucidating predictions by assessing the impact of individual features. This method calculates average Shapley values using game theory principles, clarifying predictions through the contribution of each data feature.

(12) $$\phi_{i}=\sum_{S\subseteq N\setminus \left\{i\right\}}\frac{\left|S\right|!\left(M-\left|S\right|-1\right)!}{M!}[f_{x}\left(S\cup \left\{i\right\}\right)-f_{x}(S)]$$

$\phi_{i}$ is the Shapley value for the data, and N is the set of total input variables. S is the set of variables except for the i-th variable in the total input variable, and v(S) is the contribution that the remaining subset, except the i-th data, contributed to the result, and $f_{x}(S\cup \left\{i\right\})$ is the total contribution including the i-th data.

In this study, we also generated equally weighted soft-voting regressors to check the average overall performance and analyzed their mean absolute SHAP values.

(13) $$I_{j}=\frac{1}{N}\sum_{i=1}^{N}\left|\phi_{j}^{\left(i\right)}\right|$$

#### 3.5.5. Performance Metrics of Classification Problem

In addition, we calculated the relationship between the prediction values and real values (fluctuation) using the confusion matrix derived from the classification results. The confusion matrix is typically used to ascertain whether the predicted value was derived appropriately compared with the actual value. In this experiment, the confusion matrix was used to determine the extent to which up or down predictions fall into the fluctuations of U.S. sector index ETFs’ prices.

**Figure 2:**
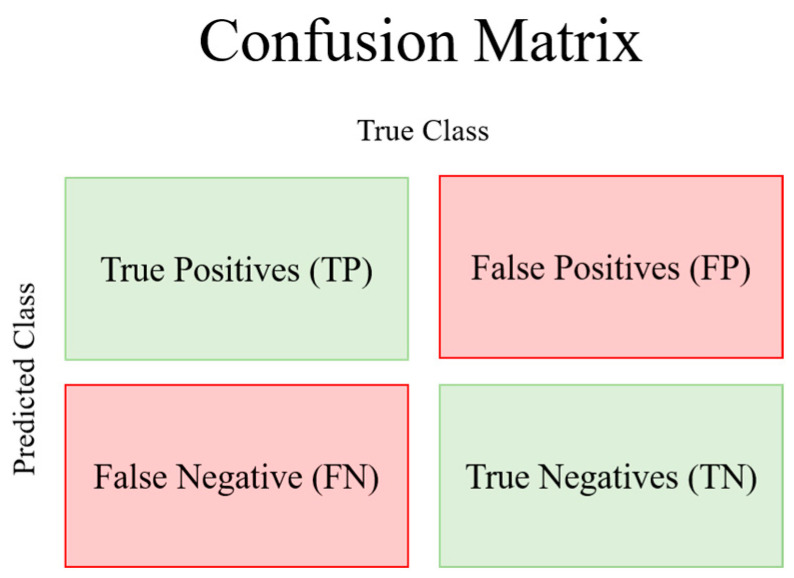
Confusion matrix.

Figure 2 shows a confusion matrix, and Table 3 further presents the evaluation metric used for the confusion matrix.

## 4. Results

### 4.1. Exploratory Data Analysis (EDA)

Table 4 and Table 5 present the descriptive statistics for our two datasets, focusing on log return and the rate of change in trading volume for various securities like XLB, XLE, XLF, and others. For the price data in Table 4, the mean values indicate the average return of each security over the studied timeframe. Most returns are proximate to zero, but XLK and XLY stand out with the highest mean returns of 0.0006. The standard deviation showcases the inherent volatility or risk, with XLE being the most volatile, having a standard deviation of 0.0183, and XLP being the least volatile at 0.0090. The minimum and maximum values capture the extreme returns; XLE saw the largest negative return at −0.2249, while XLF recorded the highest positive return at 0.1524. Quartiles, particularly the medians, often reveal positive returns aligned with the mean values. The skewness of most securities is negative, suggesting that the left tail, or the negative returns, extends more than the right. The kurtosis, which is consistently greater than 3 for all securities, points to a distribution with heavier tails than a normal distribution, implying a higher likelihood of extreme values or outliers.

In Table 5, which covers trading volume data, we observe that XLF, intriguingly, has a negative mean, hinting at a general decreasing trend in its trading volume. On the volatility front, XLP leads with the most volatile trading volume. The skewness values for volume differ across securities, suggesting varied asymmetry in their volume distributions, and the kurtosis indicates that spikes or dramatic drops in trading volumes can occasionally occur.

Several statistical tests were performed, such as the Shapiro–Wilk test, Kolmogorov–Smirnov test, and Jarque–Bera test for normality. The Ljung–Box test was employed to scrutinize the autocorrelation at different orders, and the Augmented Dickey–Fuller (ADF) test was used to check for stationarity. The symbols *, **, and *** denote statistical significance at the 0.1, 0.05, and 0.01 levels. The outcome of this rigorous testing reveals that none of the data columns adhere to normality across both sets of descriptive statistics. This non-compliance with normality furnishes a quantitative foundation for leveraging nonparametric methodologies that do not rest on assumptions like normality.

For the more detailed analysis of non-normality in Table 4 and Table 5, Table 6 and Table 7 delineate the ratios at which the null hypothesis was rejected during normality testing for all generated datasets. Seven normality tests were conducted, including the Shapiro–Wilk test, D’Agostino K-squared test, and others. A striking revelation from these tests is that more than 80% of the dataset used for calculations failed to meet the criteria for normality. This discovery underpins our methodology decision to employ nonlinear nonparametric measures like mutual information and transfer entropy, which remains unfazed by normality prerequisites. The central limit theorem (CLT) theoretically suggests that as the sample size swells, the distribution of sample means should approximate a Gaussian distribution. However, our empirical findings, through exploratory data analysis (EDA), demonstrate a significant trend: as the window length elongates, the null hypothesis becomes rejected more frequently across all normality tests.

### 4.2. Prediction Results

#### 4.2.1. Prediction Performance

We conducted 100 iterations, changing their seeds to consider the robustness of our experiments. Table 8 shows the average values of our predictions’ performance metrics. The original dataset includes U.S. sector index ETFs’ price-related data.

In our comprehensive analysis, meticulous care was taken to ensure the robustness of our experimental results. To this end, a series of 100 iterations were conducted, each employing a distinct seed, thereby enhancing the reliability and generalizability of our findings.

Presented in Table 8 are the aggregate values derived from our prediction’s performance metrics. This table facilitates a nuanced comparative examination between the performance outcomes obtained from the original dataset, predominantly price-related data from U.S. sector index ETFs, and the outcomes following the integration of advanced features. Specifically, these newly incorporated features are grounded in mutual information (MI) and transfer entropy (TE)-based network embeddings coupled with intricate network topology measures.

The last three columns of Table 8 are dedicated to the Paired T-test results. In evaluating our models, we employed a paired *t*-test to statistically validate the observed differences between the mean values obtained using different methods. The paired *t*-test was executed on three machine learning techniques’ results. Depending on the specific category under consideration, we adjusted our hypothesis. For most categories, our alternative hypothesis posited that the mean values in columns like the performance metrics, after including MI and TE network-based columns (network embeddings and network measures), were greater than their corresponding values in the original datasets, respectively. However, for the “Hamming Loss” category, we reversed this hypothesis, testing if the mean values in the original datasets’ performance metrics were greater than those in the performance metrics after including MI and TE network-based columns.

Our results, formatted with significance levels, clearly indicated the differences between the paired columns. Significance levels were denoted with asterisks, where ‘***‘ indicates a 0.01 level significance, ‘**‘ indicates a 0.05 level, and ‘*‘ indicates a 0.1 level. The values with a *p*-value greater than 0.1 are presented without any asterisks.

In Table 8, a comprehensive evaluation of three machine learning models—XGBoost, LightGBM, and CatBoost—is presented across various metrics and datasets. The datasets under consideration include the original dataset and a refined dataset enriched with Proposed features, specifically the MI and TE-based network-driven features. The table also highlights the results of the paired *t*-test statistic, which provides insight into the statistical significance of the performance differences observed between the two datasets.

For each sector, such as XLB (Materials) and XLE (Energy), we observed the performance of the three models on both datasets. At a glance, it is evident that the dataset with proposed features often achieves better or comparable results than the original dataset across most sectors and metrics. This indicates that the added features provide valuable information that enhances the model’s performance.

The XGBoost model consistently demonstrates improved performance on the dataset with the proposed features across nearly all sectors. The improvements are especially noticeable in sectors like XLE (Energy) and XLK (Technology), with the significance of this observed improvement reinforced by the paired *t*-test statistic.

LightGBM, on the other hand, while benefiting from the proposed features in sectors like XLB (Materials) and XLV (Health Care), shows diminished performance in others, such as the XLI (Industrials) sector. This suggests that while the proposed features enhance model robustness, they might introduce noise or redundancy for specific models or sectors.

CatBoost’s performance, compared to the other two models, is generally superior on the proposed dataset, especially in sectors like XLB (Materials) and XLK (Technology). The *t*-test values further validate the significance of these observations.

Diving deeper into the metrics, accuracy, which provides a general sense of model performance, often shows noticeable improvement when models are trained on the dataset with proposed features. Similarly, balanced accuracy, which provides a more nuanced view, especially in imbalanced datasets, mirrors the trends of regular accuracy. Cohen’s Kappa Coefficient, which assesses the agreement between predicted and actual classifications, significantly improves models like XGBoost in sectors such as XLE (Energy).

Other metrics, such as precision, recall, F1, and F-Beta scores, provide a detailed view of model performance by considering false positives and false negatives. Across most sectors, the dataset with proposed features tends to enhance these metrics for all three models, especially for XGBoost and CatBoost. The Hamming Loss metric, which evaluates the fraction of incorrectly predicted labels, also indicates fewer incorrect predictions for many sectors when using the proposed features.

In conclusion, Table 8 underscores the potential of the proposed MI and TE-based network-driven features in enhancing machine learning model performance across various sectors. While all three models benefit from these features, XGBoost and CatBoost often show the most pronounced improvements. The paired *t*-test statistics emphasize the value and reliability of incorporating the proposed features into the dataset.

#### 4.2.2. Feature Importance of Causal Network-Related Features

We used the mean absolute SHAP values from the prediction results to elucidate the impact of our causal network-derived measures. Table 9, Table 10, Table 11, Table 12, Table 13 and Table 14 detail the top 20 features determined by the mean absolute SHAP values from the prediction results. The decision to use the top 20 features was guided by an elbow method, revealing a notable difference around the twentieth feature. Therefore, for our nine target ETFs across three gradient boosting algorithms (XGBoost, LightGBM, and CatBoost), a total of 540 features are presented. In detail, 20 (top 20 important features derived from mean absolute SHAP values) × 3 (the number of gradient boosting algorithms) × 9 (the number of target ETFs) features were used for the post-xAI analysis. We conducted a comprehensive analysis of the 540 features in our dataset. These features were systematically categorized to understand their characteristics and relationships better. By grouping them, we could identify patterns, trends, and anomalies more clearly, ensuring a streamlined and efficient data assessment.

In Table 9, of the 540 features, our proposed features constitute 283 (52.41%), while the original features account for 257 (47.59%). Given that the 18 original features are composed of nine log-return columns and nine columns depicting the rate of change in the trading volume, it implies that one feature, on average, appears approximately 14.3 times across the 27 models. Notably, the trading volume contributes nearly as much as price, solidifying its relevance in our study.

Table 10 underscores that roughly 70% of node-derived features stem from the MI network, encapsulating over a third (36.67%) of the 540 features. This suggests the importance of nonlinear mutual dependencies in analyzing the U.S. sector index. Notably, though nonlinear causal relationships are not ubiquitous, they hold significance when they emerge, especially for fluctuation prediction.

Observations from Table 11 demonstrate that features derived from 20-day, 60-day, 120-day, and 240-day windows span short-term to long-term influences on the U.S. sector index ETFs’ fluctuations. Short-term (20-day) features particularly exert substantial influence, more so with trading volume data than price-based log-return data. This indicates that nonlinear dependencies and causality hold consistent importance in gradient-boosting algorithm-based predictions.

Our findings, as detailed in Table 12, indicate that centrality measures and node embeddings both play pivotal roles in predicting U.S. sector index ETFs. Most notably, of the 32 node embedding-based features, 130 make the top 20 based on the mean absolute SHAP values, implying that node embeddings are instrumental in enhancing prediction accuracy.

Table 13 highlights that nearly all centrality measures are among the top 20 in predicting fluctuations in U.S. sector index ETFs. Specifically, second-order centrality, PageRank, and HITS—which all consider relative connections as opposed to direct ones—are predominant. This supports our approach of emphasizing interconnectivity and causality through network analysis.

Conclusively, in Table 14, while Role2vec (a structural node embedding algorithm) represents 56.92% of node embeddings, FEATHER (an attributed node embedding algorithm) is also notable at 43.08%. This paves the way for exploring a broader range of node embeddings in subsequent studies. Additionally, refining the embedding vector dimensions merits further investigation, given its variability based on objectives.

## 5. Discussion

To understand the intricacies of U.S. sector index ETFs’ price fluctuations, this study embarked on a mission to validate the existence and implications of nonlinear dependencies and causal relationships emanating from log returns and the rate of changes in trading volumes. By incorporating sophisticated techniques such as mutual information, transfer entropy with permutation tests, and threshold networks, we sought insights from the intricate web of relationships that govern ETFs’ price dynamics. Our methodological approach led to the construction of undirected and directed weighted networks, illuminating the nonlinear dependencies and causal dynamics between the U.S. sector index ETFs.

A significant revelation of our research was the potential to enhance the predictive prowess of machine learning models using centrality measures and node embeddings derived from the topology of these constructed networks. By integrating these measures into our prediction models, not only did we achieve superior forecasting accuracy, but we also heightened the explanatory capabilities of our models. The visual representation of information-theoretic dependency and information transfer through the networks further underscored the relationships and dependencies between the ETFs, offering a richer understanding of their interconnected dynamics.

A cornerstone of our analysis was using SHAP, rooted in Shapley values, to quantify the effectiveness of our centrality measures and node embeddings in forecasting the returns of U.S. sector index ETFs. The insights gleaned from analyzing the mean of absolute SHAP values were revelatory. They affirmed that features derived from our information-theoretic networks, especially those grounded in various temporal windows ranging from short-term (20-day) to long-term (240-day) windows, were pivotal in forecasting the fluctuations of U.S. sector index futures. This not only bolstered the case for employing log returns and trading volumes’ rates of changes as reliable measures for capturing mutual information and transferring entropy across diverse temporal windows, but also solidified their utility in crafting a plethora of networks and subsequently harnessing their node-level properties.

Our study illuminated the path forward in forecasting U.S. sector index futures. By harnessing the power of nonlinear measure-based networks and their node-derived features, we not only refined the predictive accuracy of our models but also enriched their explainability. This study’s revelations underscore these techniques’ promises and set the stage for future explorations in this domain.

## 6. Conclusions

In our quest to understand and predict the dynamics of U.S. stock market sector indices, our study introduced a unique perspective by employing a nonlinear, nonparametric measure-based complex network approach. Harnessing the nuanced insights from information entropy-based measures, we shed light on the intricate information-theoretic relationships embedded within U.S. stock market sector indices’ price and trading volume data. By delving into these nonlinear dependencies and causal relationships, we ventured into an under-explored domain and offered a fresh lens for forecasting U.S. sector index ETF prices.

Some of the distinct contributions of our study include the following:

- The utilization of information entropy-based measures to discern and showcase the underlying relationships in U.S. stock market sector indices.
- The illustration of nonlinear dependencies and causal relationships in the U.S. market sector index networks, which is an area that has yet to be deeply probed.
- The revelation that nonlinear dependencies and causal relationships can significantly contribute to predictive models, shedding light on new avenues in market forecasting.
- Empirical evidence supports using return-based data to bolster prediction results by probing into the intricate webs formed by return and trading volume networks. This offers a promising direction in enhancing data efficiency by leveraging inter-sectoral relationships without additional external features.

Despite our contribution, we recognize that there is always room for growth and improvement. For starters, diving deeper into the vast world of machine learning and graph embedding techniques could sharpen our analysis. These advanced tools might offer a more transparent lens to view our predictions. We also acknowledge that perfecting our models and tweaking their internal settings or “hyperparameters” could potentially enhance the accuracy of our sector forecasts. Moreover, while our focus has been specific, bringing in data like macroeconomic variables could offer a fuller picture and add depth to our findings.

While our research has added value to the financial sector’s understanding, it is just one step in a longer journey. We are excited about future studies and the potential to delve even deeper into the intricacies of financial forecasting.

## Figures and Tables

**Table 1:** Sector ETFs and their descriptions.

Full Name	Abbreviation	Tracking Index	Specialized Description
Materials	XLB	S&P Materials Index	Tracks companies in the materials sector, including chemicals, mining, and forestry.
Energy	XLE	S&P Energy Index	Comprises companies in the energy sector, including oil, gas, and renewable energy.
Financials	XLF	S&P Financials Index	Represents companies in the financial sector, including banks, insurance, and real estate services.
Industrials	XLI	S&P Industrials Index	Covers companies in the industrial sector, such as machinery, aerospace, and defense.
Technology	XLK	S&P Technology Index	Represents the technology sector, including software, hardware, and electronics.
Consumer Staples	XLP	S&P Consumer Staples Index	Consists of companies in the consumer staples sector, like food, beverages, and household goods.
Utilities	XLU	S&P Utilities Index	Represents utilities sector companies, including electric, gas, and water utilities.
Health Care	XLV	S&P Health Care Index	Covers companies in the health care sector, including pharmaceuticals, biotech, and health care services.
Consumer Discretionary	XLY	S&P Consumer Discretionary Index	Represents companies in the consumer discretionary sector, like entertainment, retail, and autos.

**Table 2:** Centrality measures and their definitions.

Centrality Measures	Citation
**Average Neighbor Degree**	[50,60]
**Degree Centrality**	[60]
**Eigenvector Centrality**	[44,60,68]
**Closeness Centrality**	[43,46,60]
**Information Centrality**	[45,60,65]
**Betweenness Centrality**	[42,49,57,58,60]
**Current Flow Betweenness Centrality**	[53,55,60]
**Communicability Betweenness Centrality**	[60,61]
**Harmonic Centrality**	[60,64]
**Second-Order Centrality**	[60,62]
**Voterank Importance**	[60,66]
**Number of Maximal Cliques**	[41,51,59]
**PageRank**	[48,60]
**HITS**	[47,54,60]

**Table 3:** Performance metrics of classification problems.

Metric	Definition
**Accuracy**	$\frac{TP\;+\;TN}{P\;+\;N}$
**Balanced Accuracy**	$\frac{TP/P\;+\;TN/N}{2}$
**Cohen’s Kappa Coefficient**	$\frac{2\;\times \;(TP\;\times \;TN-FN\;\times \;FP)}{\left(TP\;+\;FP\right)\;\times \;\left(FP\;+\;TN\right)\;+\;(TP\;+\;FN)\;\times \;(FN+TN)}$
**Precision**	$\frac{TP}{TP\;+\;FP}$
**Recall**	$\frac{TP}{TP+FN}$
**F1 Score**	$\frac{2TP}{2TP\;+\;FP\;+\;FN}$
**F-Beta Score**	$\frac{(1\;+\;\beta^{2})\;\times \;Precision\;\times \;Recall}{\beta^{2}\;\times \;Precision+Recall}$
**Hamming Loss**	$\frac{1}{n_{samples}\times n_{labels}}\sum_{i=0}^{n_{samples}-1}\sum_{j=0}^{n_{labels}-1}1\left({\hat{y}}_{i,j}\neq y_{i,j}\right)$ where ${\hat{y}}_{i,j}$ is the predicted value for the j-th label of a given sample i, $y_{i,j}$ is the corresponding true value, $n_{samples}$ is the number of samples, $n_{labels}$ is the number of labels (in this study, $n_{labels}=2)$, and 1(x) is the indicator function.

**Table 4:** Descriptive statistics (price).

Statistics	XLB	XLE	XLF	XLI	XLK	XLP	XLU	XLV	XLY
**Mean**	0.0004	0.0002	0.0004	0.0004	0.0006	0.0004	0.0004	0.0005	0.0006
**Standard Deviation**	0.0143	0.0183	0.0177	0.0134	0.0135	0.0090	0.0112	0.0107	0.0132
**Min.**	−0.1166	−0.2249	−0.1807	−0.1204	−0.1487	−0.0987	−0.1206	−0.1038	−0.1355
**Max.**	0.1112	0.1487	0.1524	0.1191	0.1109	0.0817	0.1204	0.0742	0.0897
**Q1**	−0.0063	−0.0078	−0.0065	−0.0054	−0.0049	−0.0036	−0.0051	−0.0044	−0.0050
**Median**	0.0009	0.0004	0.0007	0.0009	0.0011	0.0007	0.0009	0.0009	0.0013
**Q3**	0.0080	0.0090	0.0079	0.0069	0.0072	0.0051	0.0062	0.0062	0.0072
**Skewness**	−0.4846	−0.7722	−0.0455	−0.4728	−0.5109	−0.5013	−0.3185	−0.4312	−0.6604
**Kurtosis**	6.0502	13.9521	16.4934	8.9646	9.7817	13.0281	16.1477	7.4502	7.9877
**Shapiro–Wilk Test (W)**	0.9421 ***	0.9073 ***	0.837 ***	0.9144 ***	0.9175 ***	0.9033 ***	0.8903 ***	0.9321 ***	0.9227 ***
**Jarque–Bera Test (JB)**	5395.7801 ***	28,327.5218 ***	39,109.1785 ***	11,680.1006 ***	13,903.6934 ***	24,544.2796 ***	37,543.7296 ***	8084.7764 ***	9421.6981 ***
**Augmented Dickey–Fuller (** ${DF}_{\tau }$ **)**	−14.9626 ***	−11.5752 ***	−10.9766 ***	−11.6973 ***	−12.9465 ***	−18.7009 ***	−14.6622 ***	−16.2154 ***	−11.9238 ***
**Ljung–Box Test (Q(5))**	27.8087 ***	21.2132 ***	100.1074 ***	22.9614 ***	70.5875 ***	59.3114 ***	61.5096 ***	42.4347 ***	23.582 ***
**Ljung–Box Test (Q(10))**	65.3377 ***	60.4795 ***	164.8877 ***	83.7618 ***	190.4879 ***	142.5232 ***	154.016 ***	121.0344 ***	78.3964 ***
**Ljung–Box Test (Q(20))**	87.2127 ***	86.7658 ***	207.5788 ***	107.8259 ***	237.6269 ***	162.8771 ***	209.2207 ***	161.026 ***	100.1244 ***

**Table 5:** Descriptive statistics (volume).

	Data	XLB	XLE	XLF	XLI	XLK	XLP	XLU	XLV	XLY
Statistics										
**Mean**		0.0002	0.0000	−0.0003	0.0001	0.0002	0.0005	0.0005	0.0005	0.0001
**Standard Deviation**		0.3912	0.3271	0.3676	0.3730	0.4187	0.4396	0.3918	0.3958	0.4108
**Min.**		−1.5326	−1.5394	−1.3862	−1.8871	−1.8236	−1.7922	−1.5858	−2.1560	−1.6051
**Max.**		1.7235	1.3046	1.5283	1.5598	1.9678	2.4972	1.6630	1.9397	2.1229
**Q1**		−0.2523	−0.2091	−0.2497	−0.2495	−0.2570	−0.2753	−0.2428	−0.2586	−0.2596
**Median**		−0.0080	−0.0093	−0.0059	0.0026	−0.0064	−0.0130	−0.0112	−0.0114	−0.0068
**Q3**		0.2471	0.2055	0.2293	0.2357	0.2504	0.2534	0.2452	0.2376	0.2450
**Skewness**		0.1301	0.0791	0.1892	0.0980	0.0656	0.2973	0.1710	0.0994	0.1642
**Kurtosis**		0.7003	0.6145	0.4871	0.7279	1.2428	1.6248	0.8267	0.9842	1.1379
**Shapiro–Wilk Test (W)**		0.9956 ***	0.996 ***	0.9963 ***	0.9956 ***	0.9906 ***	0.9857 ***	0.9935 ***	0.9935 ***	0.9918 ***
**Jarque–Bera Test (JB)**		79.9351 ***	57.6037 ***	54.5113 ***	81.3655 ***	223.9659 ***	429.7603 ***	114.7139 ***	144.4836 ***	201.1662 ***
**Augmented Dickey–Fuller** (${DF}_{\tau }$)		−20.5462 ***	−15.9105 ***	−15.1273 ***	−15.3356 ***	−16.3168 ***	−16.3461 ***	−16.415 ***	−19.7349 ***	−20.9397 ***
**Ljung–Box Test (Q(5))**		439.3993 ***	438.0747 ***	427.4039 ***	442.3769 ***	545.3189 ***	598.9662 ***	632.7569 ***	483.8212 ***	594.0558 ***
**Ljung–Box Test (Q(10))**		442.1597 ***	456.3206 ***	431.9172 ***	445.1095 ***	547.6234 ***	618.9684 ***	639.1071 ***	489.3571 ***	599.5767 ***
**Ljung–Box Test (Q(20))**		473.9014 ***	482.8198 ***	458.9153 ***	464.1181 ***	558.6023 ***	647.9025 ***	654.0895 ***	494.3574 ***	610.6616 ***

**Table 6:** Rejected ratios of all generated datasets derived from the normality test results (price).

(Unit: %)	Shapiro–Wilk Test (W)	D’Agostino K-Squared Test (K-Squared)	Lilliefors Test (T)	Jarque–Bera Test (JB)	Kolmogorov–Smirnov Test (KS)	Anderson–Darling Test (A-Squared)	Cramér–von Mises Test (U)
**XLB**							
***α* = 0.1**	91.69	91.54	89.33	91.54	100.00	92.39	100.00
***α* = 0.05**	90.28	90.05	87.01	90.53	100.00	90.60	100.00
***α* = 0.01**	87.30	87.23	82.46	88.43	100.00	87.32	100.00
**XLE**							
***α* = 0.1**	93.41	92.72	89.89	92.81	100.00	92.98	100.00
***α* = 0.05**	92.27	91.64	87.66	92.10	100.00	91.22	100.00
***α* = 0.01**	90.46	89.92	83.74	90.94	100.00	87.88	100.00
**XLF**							
***α* = 0.1**	94.11	93.99	91.96	93.99	100.00	93.94	100.00
***α* = 0.05**	93.04	92.75	90.33	93.16	100.00	92.37	100.00
***α* = 0.01**	90.49	89.69	87.36	91.39	100.00	89.93	100.00
**XLI**							
***α* = 0.1**	93.34	93.30	92.48	93.43	100.00	94.14	100.00
***α* = 0.05**	92.01	91.97	90.58	92.58	100.00	92.86	100.00
***α* = 0.01**	89.69	89.56	86.69	90.93	100.00	89.91	100.00
**XLK**							
***α* = 0.1**	95.88	95.65	93.83	95.51	100.00	95.94	100.00
***α* = 0.05**	94.89	94.69	91.89	94.83	100.00	94.80	100.00
***α* = 0.01**	92.62	92.44	88.36	93.51	100.00	92.41	100.00
**XLP**							
***α* = 0.1**	94.47	94.86	91.74	94.80	100.00	94.47	100.00
***α* = 0.05**	93.13	93.81	89.40	94.15	100.00	92.89	100.00
***α* = 0.01**	90.86	91.86	84.82	92.85	100.00	89.68	100.00
**XLU**							
***α* = 0.1**	90.83	91.65	88.12	91.65	100.00	89.81	100.00
***α* = 0.05**	89.13	89.98	85.62	90.46	100.00	88.06	100.00
***α* = 0.01**	86.40	87.49	81.01	88.65	100.00	84.48	100.00
**XLV**							
***α* = 0.1**	93.61	93.91	90.51	93.85	100.00	93.76	100.00
***α* = 0.05**	92.55	93.06	87.93	93.3	100.00	92.30	100.00
***α* = 0.01**	89.75	90.59	83.98	91.91	100.00	88.77	100.00
**XLY**							
***α* = 0.1**	95.22	95.48	92.54	95.19	100.00	94.53	100.00
***α* = 0.05**	94.00	94.37	90.48	94.39	100.00	93.41	100.00
***α* = 0.01**	91.83	91.83	86.04	92.69	100.00	91.05	100.00

**Table 7:** Rejected ratios of all generated datasets derived from the normality test results (volume).

(Unit: %)	Shapiro–Wilk Test (W)	D’Agostino K-Squared Test (K-Squared)	Lilliefors Test (T)	Jarque–Bera Test (JB)	Kolmogorov–Smirnov Test (KS)	Anderson–Darling Test (A-Squared)	Cramér–von Mises Test (U)
**XLB**							
***α* = 0.1**	75.04	77.25	40.81	77.12	99.78	68.89	99.90
***α* = 0.05**	70.16	73.57	25.55	74.01	99.54	61.10	99.75
***α* = 0.01**	60.57	65.45	1.87	68.97	98.74	46.41	99.12
**XLE**							
***α* = 0.1**	76.65	79.49	37.70	79.47	99.95	64.88	99.99
***α* = 0.05**	69.92	73.76	27.75	75.62	99.84	54.95	99.94
***α* = 0.01**	50.64	50.83	11.27	62.36	99.34	35.56	99.56
**XLF**							
***α* = 0.1**	78.19	79.61	51.59	80.01	99.89	70.32	99.96
***α* = 0.05**	73.37	74.10	42.80	75.08	99.71	63.23	99.86
***α* = 0.01**	61.00	63.86	25.21	66.37	99.06	50.93	99.31
**XLI**							
***α* = 0.1**	73.71	76.71	28.21	77.52	99.88	49.85	99.95
***α* = 0.05**	70.07	73.61	16.67	75.21	99.66	38.19	99.84
***α* = 0.01**	56.83	67.41	2.09	71.26	99.00	15.81	99.28
**XLK**							
***α* = 0.1**	85.99	86.03	77.41	86.33	99.73	85.93	99.87
***α* = 0.05**	83.65	83.98	69.76	85.21	99.46	81.83	99.68
***α* = 0.01**	78.64	80.26	54.79	82.91	98.62	74.94	99.00
**XLP**							
***α* = 0.1**	87.34	89.86	79.01	89.63	99.57	85.64	99.75
***α* = 0.05**	84.44	86.88	74.78	87.34	99.24	82.04	99.52
***α* = 0.01**	78.91	80.89	66.92	82.93	98.25	73.63	98.74
**XLU**							
***α* = 0.1**	79.74	83.58	68.10	84.63	99.78	77.19	99.89
***α* = 0.05**	74.97	78.16	62.55	80.92	99.56	72.42	99.74
***α* = 0.01**	64.45	64.73	47.43	71.40	98.80	62.06	99.15
**XLV**							
***α* = 0.1**	83.72	85.21	64.76	85.86	99.80	82.53	99.91
***α* = 0.05**	79.20	80.76	55.61	82.49	99.58	77.04	99.75
***α* = 0.01**	68.36	69.73	43.06	75.57	98.84	62.80	99.13
**XLY**							
***α* = 0.1**	73.54	77.90	57.74	78.33	99.74	70.27	99.86
***α* = 0.05**	68.80	73.29	50.16	74.77	99.48	63.39	99.67
***α* = 0.01**	62.41	66.20	31.03	69.55	98.64	53.53	98.98

**Table 8:** Prediction results.

Model	Original Dataset			Dataset with Proposed Features (MI and TE-Based Network-Driven Features)			Independent *t*-Test Statistic		
	XGBoost	LightGBM	CatBoost	XGBoost	LightGBM	CatBoost	XGBoost	LightGBM	CatBoost
**XLB (Materials)**									
**Accuracy**	0.5376	0.5418	0.5312	0.5460	0.5460	0.5619	14.17 ***	7.03 ***	63.38 ***
**Balanced Accuracy**	0.5389	0.5431	0.5324	0.5454	0.5426	0.5594	15.41 ***	−2.11	64.49 ***
**Cohen’s Kappa Coefficient**	0.0773	0.0857	0.0644	0.0906	0.0855	0.1190	297.86 ***	−2.31	711.19 ***
**Precision**	0.5408	0.5451	0.5343	0.5471	0.5446	0.5612	11.61 ***	−1.03	109.54 ***
**Recall**	0.5376	0.5418	0.5312	0.5460	0.5460	0.5619	14.49 ***	6.35 ***	59.31 ***
**F1 Score**	0.5379	0.5421	0.5315	0.5464	0.5450	0.5615	18.34 ***	8.83 ***	67.71 ***
**F-Beta Score (0.5)**	0.5393	0.5435	0.5329	0.5468	0.5447	0.5613	14.90 ***	1.78 **	80.04 ***
**F-Beta Score (2)**	0.5374	0.5416	0.5311	0.5464	0.5450	0.5615	22.20 ***	6.79 ***	65.44 ***
**Hamming Loss**	0.4624	0.4582	0.4688	0.4540	0.4540	0.4381	19.40 ***	8.59 ***	72.07 ***
**XLE (Energy)**									
**Accuracy**	0.5238	0.5270	0.5439	0.5651	0.5503	0.5365	103.61 ***	38.95 ***	−14.99
**Balanced Accuracy**	0.5263	0.5307	0.5485	0.5578	0.5422	0.5286	52.75 ***	36.89 ***	−34.53
**Cohen’s Kappa Coefficient**	0.0523	0.0609	0.0960	0.1173	0.0857	0.0580	1282.24 ***	389.73 ***	−717.6
**Precision**	0.5275	0.5328	0.5527	0.5681	0.5520	0.5352	67.81 ***	41.47 ***	−52.66
**Recall**	0.5238	0.5270	0.5439	0.5651	0.5503	0.5365	88.46 ***	38.01 ***	−9.78
**F1 Score**	0.5219	0.5225	0.5372	0.5475	0.5277	0.5139	57.68 ***	10.00 ***	−70.39
**F-Beta Score (0.5)**	0.5243	0.5268	0.5436	0.5545	0.5354	0.5203	161.22 ***	11.99 ***	−38.32
**F-Beta Score (2)**	0.5221	0.5235	0.5387	0.5539	0.5363	0.5228	66.91 ***	20.64 ***	−37.53
**Hamming Loss**	0.4762	0.4730	0.4561	0.4349	0.4497	0.4635	120.90 ***	42.91 ***	−19.04
**XLF (Financials)**									
**Accuracy**	0.5259	0.5238	0.5407	0.5407	0.5249	0.5503	43.08 ***	−0.42	14.55 ***
**Balanced Accuracy**	0.5279	0.5246	0.5413	0.5362	0.5263	0.5477	18.78 ***	5.92 ***	9.85 ***
**Cohen’s Kappa Coefficient**	0.0555	0.0491	0.0823	0.0729	0.0523	0.0957	232.33 ***	62.69 ***	124.04 ***
**Precision**	0.5294	0.5259	0.5425	0.5383	0.5276	0.5491	18.96 ***	0.89	13.99 ***
**Recall**	0.5259	0.5238	0.5407	0.5407	0.5249	0.5503	32.48 ***	0.86	57.54 ***
**F1 Score**	0.5255	0.5240	0.5410	0.5370	0.5248	0.5493	18.74 ***	3.61 ***	17.84 ***
**F-Beta Score (0.5)**	0.5273	0.5249	0.5417	0.5372	0.5261	0.5491	16.38 ***	10.47 ***	6.55 ***
**F-Beta Score (2)**	0.5253	0.5237	0.5407	0.5387	0.5245	0.5498	26.20 ***	2.29 **	24.94 ***
**Hamming Loss**	0.4741	0.4762	0.4593	0.4593	0.4751	0.4497	36.54 ***	4.50 ***	34.39 ***
**XLI (Industrials)**									
**Accuracy**	0.5354	0.5164	0.5376	0.5302	0.4910	0.5450	−13.62	−90.49	33.05 ***
**Balanced Accuracy**	0.5345	0.5131	0.5334	0.5328	0.4995	0.5480	−4.78	−76.45	38.29 ***
**Cohen’s Kappa Coefficient**	0.0688	0.0262	0.0670	0.0649	−0.0009	0.0949	−83.71	−2684.93	296.78 ***
**Precision**	0.5371	0.5159	0.5362	0.5357	0.5023	0.5511	−4.06	−30.59	35.38 ***
**Recall**	0.5354	0.5164	0.5376	0.5302	0.4910	0.5450	−10.48	−109.98	14.31 ***
**F1 Score**	0.5360	0.5161	0.5367	0.5303	0.4851	0.5450	−11.23	−81.94	27.85 ***
**F-Beta Score (0.5)**	0.5366	0.5160	0.5363	0.5329	0.4924	0.5479	−7.1	−106.52	28.69 ***
**F-Beta Score (2)**	0.5356	0.5163	0.5372	0.5296	0.4860	0.5443	−8.79	−50.11	25.06 ***
**Hamming Loss**	0.4646	0.4836	0.4624	0.4698	0.5090	0.4550	−14.29	−176.44	18.14 ***
**XLK (Technology)**									
**Accuracy**	0.4899	0.5365	0.5090	0.5513	0.5333	0.5397	164.25 ***	−3.89	158.33 ***
**Balanced Accuracy**	0.4866	0.5304	0.5017	0.5342	0.5267	0.5211	112.09 ***	−5.91	36.56 ***
**Cohen’s Kappa Coefficient**	−0.0268	0.0611	0.0035	0.0703	0.0538	0.0434	1838.24 ***	−136.5	926.00 ***
**Precision**	0.4907	0.5344	0.5058	0.5426	0.5308	0.5284	179.15 ***	−15.42	78.61 ***
**Recall**	0.4899	0.5365	0.5090	0.5513	0.5333	0.5397	162.94 ***	−9.32	69.18 ***
**F1 Score**	0.4903	0.5351	0.5067	0.5345	0.5316	0.5192	97.61 ***	−5.93	40.65 ***
**F-Beta Score (0.5)**	0.4905	0.5346	0.5060	0.5360	0.5310	0.5206	108.76 ***	−6.11	22.56 ***
**F-Beta Score (2)**	0.4901	0.5359	0.5079	0.5419	0.5325	0.5283	414.04 ***	−9.23	45.26 ***
**Hamming Loss**	0.5101	0.4635	0.4910	0.4487	0.4667	0.4603	171.61 ***	−9.13	79.72 ***
**XLP (Consumer Staples)**									
**Accuracy**	0.4984	0.5185	0.5196	0.5354	0.5185	0.5429	179.05 ***	−0.8	41.66 ***
**Balanced Accuracy**	0.4941	0.5124	0.5117	0.5336	0.5130	0.5307	178.37 ***	2.05 **	41.78 ***
**Cohen’s Kappa Coefficient**	−0.0117	0.0250	0.0236	0.0670	0.0261	0.0624	5736.80 ***	105.42 ***	1548.05 ***
**Precision**	0.4980	0.5163	0.5157	0.5372	0.5168	0.5361	219.84 ***	0.41	39.55 ***
**Recall**	0.4984	0.5185	0.5196	0.5354	0.5185	0.5429	67.65 ***	−1.18	58.84 ***
**F1 Score**	0.4982	0.5170	0.5165	0.5361	0.5175	0.5343	83.44 ***	3.18 ***	40.08 ***
**F-Beta Score (0.5)**	0.4981	0.5165	0.5157	0.5367	0.5170	0.5341	88.12 ***	4.62 ***	51.29 ***
**F-Beta Score (2)**	0.4983	0.5178	0.5181	0.5357	0.5180	0.5383	138.82 ***	1.80 **	36.87 ***
**Hamming Loss**	0.5016	0.4815	0.4804	0.4646	0.4815	0.4571	129.93 ***	1.04	94.83 ***
**XLU (Utilities)**									
**Accuracy**	0.5090	0.5090	0.5164	0.5291	0.5429	0.5386	44.90 ***	85.79 ***	52.85 ***
**Balanced Accuracy**	0.5075	0.5060	0.5101	0.5240	0.5254	0.5141	33.85 ***	37.36 ***	7.19 ***
**Cohen’s Kappa Coefficient**	0.0149	0.0120	0.0203	0.0480	0.0522	0.0295	683.80 ***	1142.35 ***	985.14 ***
**Precision**	0.5120	0.5106	0.5147	0.5284	0.5327	0.5227	25.40 ***	47.61 ***	16.81 ***
**Recall**	0.5090	0.5090	0.5164	0.5291	0.5429	0.5386	51.71 ***	52.67 ***	68.31 ***
**F1 Score**	0.5100	0.5097	0.5154	0.5287	0.5277	0.5066	39.26 ***	40.01 ***	−41.61
**F-Beta Score (0.5)**	0.5111	0.5102	0.5149	0.5285	0.5280	0.5089	46.33 ***	34.48 ***	−11.1
**F-Beta Score (2)**	0.5093	0.5092	0.5160	0.5289	0.5345	0.5209	87.16 ***	69.83 ***	13.87 ***
**Hamming Loss**	0.4910	0.4910	0.4836	0.4709	0.4571	0.4614	89.99 ***	94.39 ***	41.61 ***
**XLV (Health Care)**									
**Accuracy**	0.4899	0.5037	0.5069	0.5259	0.5259	0.5450	127.26 ***	34.39 ***	106.88 ***
**Balanced Accuracy**	0.4889	0.4990	0.5044	0.5192	0.5211	0.5347	87.79 ***	50.89 ***	68.11 ***
**Cohen’s Kappa Coefficient**	−0.0222	−0.0020	0.0088	0.0387	0.0424	0.0705	2969.95 ***	1817.86 ***	6413.97 ***
**Precision**	0.4915	0.5015	0.5069	0.5220	0.5237	0.5395	71.63 ***	37.44 ***	116.81 ***
**Recall**	0.4899	0.5037	0.5069	0.5259	0.5259	0.5450	88.41 ***	170.69 ***	62.38 ***
**F1 Score**	0.4905	0.5021	0.5069	0.5220	0.5242	0.5353	92.07 ***	133.52 ***	82.12 ***
**F-Beta Score (0.5)**	0.4910	0.5017	0.5069	0.5215	0.5238	0.5360	75.33 ***	119.90 ***	56.07 ***
**F-Beta Score (2)**	0.4901	0.5030	0.5069	0.5220	0.5242	0.5353	54.62 ***	107.51 ***	53.91 ***
**Hamming Loss**	0.5101	0.4963	0.4931	0.4741	0.4741	0.4550	83.94 ***	39.56 ***	162.06 ***
**XLY (Consumer Discretionary)**									
**Accuracy**	0.5185	0.5376	0.5323	0.5365	0.5524	0.5545	44.96 ***	45.45 ***	108.49 ***
**Balanced Accuracy**	0.5141	0.5319	0.5272	0.5421	0.5534	0.5546	50.47 ***	27.77 ***	55.50 ***
**Cohen’s Kappa Coefficient**	0.0281	0.0638	0.0543	0.0823	0.1053	0.1078	786.75 ***	462.79 ***	1066.78 ***
**Precision**	0.5206	0.5381	0.5334	0.5490	0.5594	0.5604	123.30 ***	36.93 ***	60.39 ***
**Recall**	0.5185	0.5376	0.5323	0.5365	0.5524	0.5545	43.30 ***	44.46 ***	65.24 ***
**F1 Score**	0.5194	0.5378	0.5328	0.5372	0.5539	0.5560	79.35 ***	34.30 ***	63.25 ***
**F-Beta Score (0.5)**	0.5200	0.5380	0.5332	0.5429	0.5567	0.5583	45.20 ***	112.82 ***	53.28 ***
**F-Beta Score (2)**	0.5188	0.5377	0.5325	0.5372	0.5539	0.5560	51.08 ***	46.74 ***	51.19 ***
**Hamming Loss**	0.4815	0.4624	0.4677	0.4635	0.4476	0.4455	57.21 ***	45.01 ***	50.27 ***

**Table 9:** Comparison of the number of original features and proposed features.

Category	Number of Features	
	Price	Volume
**Original Features**	168 (31.11%)	89 (16.48%)
**Proposed Features**	131 (24.26%)	152 (28.15%)

**Table 10:** Comparison of the number of MI-based features and TE-based features.

Category	Number of Features
**Mutual Information**	198 (69.96%)
**Transfer Entropy**	85 (30.04%)

**Table 11:** Comparison of the number of features based on window length.

Category	Number of Features	
	Price	Volume
**20-day window**	42 (14.84%)	52 (18.37%)
**60-day window**	33 (11.66%)	62 (21.91%)
**120-day window**	21 (7.42%)	33 (11.66%)
**240-day window**	35 (12.37%)	5 (1.77%)

**Table 12:** Comparison of the number of centrality measures and node embeddings.

Category	Number of Features
**Centrality Measures**	153 (54.06%)
**Node embeddings**	130 (45.94%)

**Table 13:** Comparison of the number of features based on centrality measures.

Feature	Number of Features
**Average Neighbor Degree**	9 (5.88%)
**Degree Centrality**	1 (0.65%) Out-Degree—1 (0.65%)
**Eigenvector Centrality**	13 (8.50%)
**Closeness Centrality**	0 (0.00%)
**Information Centrality**	2 (1.31%)
**Betweenness Centrality**	9 (5.88%)
**Current Flow Betweenness Centrality**	7 (4.58%)
**Communicability Betweenness Centrality**	5 (3.27%)
**Harmonic Centrality**	3 (1.96%)
**Second-Order Centrality**	34 (22.22%)
**Voterank Importance**	0 (0.00%)
**Number of Maximal Cliques**	5 (3.27%)
**PageRank**	26 (16.99%)
**HITS**	39 (25.49%) Hub—25 (16.34%) Authority—14 (9.15%)

**Table 14:** Comparison of the number of Role2Vec vectors and FEATHER vectors.

Category	Number of Features
**Role2Vec**	74 (56.92%)
**FEATHER**	56 (43.08%)

## Data Availability

Publicly available datasets were analyzed in this study. This data can be found here: https://finance.yahoo.com/ (accessed on 1 October 2023).
